# Use of Dietary Supplements among Polish Children with Inflammatory Bowel Disease: A Two-Center Pilot Study

**DOI:** 10.3390/nu16162762

**Published:** 2024-08-19

**Authors:** Monika Maćków, Agnieszka Kozioł-Kozakowska, Magdalena Szeląg, Tomasz Pytrus, Ewa Raczkowska, Katarzyna Neubauer, Ireneusz Zawiślak, Robert Gajda, Marta Habánová, Andrzej Stawarski

**Affiliations:** 1 Department of Human Nutrition, Faculty of Biotechnology and Food Science, Wrocław University of Environmental and Life Sciences, Chełmońskiego 37, 51-630 Wrocław, Poland; monika.mackow@upwr.edu.pl (M.M.); ireneusz.zawislak@upwr.edu.pl (I.Z.); robert.gajda@upwr.edu.pl (R.G.); 2 Regional Specialist Hospital in Wrocław, Research and Development Center, 51-124 Wrocław, Poland; 3 Department of Pediatrics, Gastroenterology and Nutrition, Institute of Pediatrics, Jagiellonian University Medical College, 30-663 Cracow, Poland; agnieszka.koziol-kozakowska@uj.edu.pl; 4 2nd Department and Clinic of Paediatrics, Gastroenterology and Nutrition, Wrocław Medical University, 50-367 Wrocław, Poland; magdalena.szelag@umw.edu.pl (M.S.); tomasz.pytrus@umw.edu.pl (T.P.); andrzej.stawarski@umw.edu.pl (A.S.); 5 Department and Clinic of Gastroenterology and Hepatology, Wrocław Medical University, 50-367 Wrocław, Poland; katarzyna.neubauer@umw.edu.pl; 6 Institute of Nutrition and Genomics, Faculty of Agrobiology and Food Resources, Slovak University of Agriculture, Trieda Andreja Hlinku 2, 94976 Nitra, Slovakia; marta.habanova@uniag.sk

**Keywords:** supplementation, nutritional deficiencies, diet

## Abstract

Inflammatory bowel disease (IBD) includes Crohn’s disease (CD) and ulcerative colitis (UC). These diseases are characterized by inflammation, which may be a consequence of changes in the intestinal microbiota and lead to mineral and vitamin deficiencies. The aims of this study were to determine the level of dietary supplement intake in children with IBD and to determine the influence of factors such as sex, nutritional status, diet, and other comorbidities on supplement intake. The study was conducted from May 2022 to September 2023 and was a prospective study. The group of children with IBD that ultimately qualified for the study numbered 96, and the control group numbered 30. The children who participated in the study were aged 4–18 years. Most parents of children with IBD (81.4%) declared that they use supplementation for their children, while 75% of parents of children without IBD declared giving their children nutritional supplements. Vitamins in both groups were most often given to children as dietary supplements (*p* = 0.018), including vitamin D. Depending on the diet used, the intake of vitamin B6 (*p* = 0.018), vitamin E (*p* = 0.040) and iron (*p* = 0.006) was significantly different among children with IBD. Statistical significance (*p* = 0.021) was observed for supplementation use and disease stage among children with IBD. For 80.2% of parents of children with IBD, the main reason for using supplements was a doctor’s recommendation. In the control group, 43.3% of parents indicated that the main reason for using supplements was to correct nutritional deficiencies. Supplementation was common in both groups, but attention should be paid to other current diseases in children with IBD and to nutritional status. In our opinion, educating parents about supplementation is important, especially among parents of children with IBD.

## 1. Introduction

Inflammatory bowel disease (IBD) is a chronic, debilitating gastrointestinal disease that primarily affects the adult population, although an estimated 25% of patients are under the age of 20 years [1]. The incidence and prevalence of IBD in children is highest in Northern Europe and North America and lowest in Southern Europe, Asia and the Middle East [2].

Moreover, in recent years, the age profile of IBD patients has been changing and the incidence in the pediatric population is increasing. IBD is a systemic disease that includes two main forms—Crohn’s disease (CD) and ulcerative colitis (UC)—which differ in the extent of inflammatory changes in the gastrointestinal tract, and thus in the clinical picture and spectrum of complications [1]. CD is characterized by transmural inflammation involving any part of the gastrointestinal tract. In contrast, UC typically involves superficial inflammation of the rectum extending into the adjacent mucosa to further segments of the large intestine [3]. One of the methods of nutritional treatment for CD is the CDED diet. In CD, the CDED diet is used, which is a diet consisting of whole foods combined with PEN, which is intended to help induce and maintain remission [4].

IBD patients are at a particular risk of malnutrition due to an inadequate energy supply, the implementation of an elimination diet, the occurrence of diarrhea, the presence of fistulae, surgical resections of the small intestine and other treatments [5,6,7,8,9,10,11,12]. There is a risk of malnutrition among children with IBD [13], and it may occur as often as in adults with IBD due to disease activity, low albumin levels [14] or an improperly balanced diet [15]. In a study conducted in Canada [14], the percentage of children with undernutrition was about 14.3%. Malnutrition in childhood CD is common at diagnosis and may persist despite treatment of the disease. Children with UC are also at risk of poor nutrition, but nutritional deficits may not be immediately obvious when assessing height and weight exclusively [7]. 

Pediatric patients with active IBD are at a higher risk for micronutrient deficiencies through several different mechanisms, including suboptimal oral intake, nutrient malabsorption, increased intestinal losses, systemic inflammation, a hypermetabolic state and adverse events from medication [16,17,18]. One study compared deficiencies of individual components occurring in children with CD and UC and found that iron, zinc and vitamin D deficiencies are relatively common at diagnosis, with only a limited improvement during observation. Folic acid deficiency was rare but significant; therefore, the authors recommend monitoring it periodically. Magnesium and vitamin B12 deficiencies were also present but rare [19]. Another study observed the following prevalence of deficiencies in 165 children with CD and UC, respectively: iron (56% and 27%), zinc (10% and 6%), vitamin D (22 and 13%), vitamin A (25% and 25%), vitamin E (5% and 4%), selenium (10 and 7%), copper (17% and 27%), vitamin B12 (2% and 5%) and folic acid in red blood cells (1% and 17%) [20].

In accordance with Directive 2002/46/EC, dietary supplements are “foodstuffs the purpose of which is to supplement the normal diet and which are concentrated sources of nutrients or other substances with a nutritional or physiological effect, alone or in combination, marketed in dose form, namely forms such as capsules, pastilles, tablets, pills and other similar forms, sachets of powder, ampoules of liquids, drop dispensing bottles, and other similar forms of liquids and powders designed to be taken in measured small unit quantities”. The Directive defines nutrients as vitamins and minerals [21].

According to ECCO (European Crohn’s and Colitis Organisation), patients with inflammatory bowel disease and iron deficiency anemia should take iron supplements to normalise haemoglobin levels and iron stores [22]. ESPGHAN (the European Society of Pediatric Gastroenterology, Hepatology and Nutrition) recommends vitamin D supplementation in children with IBD when there is vitamin D deficiency (i.e., a 25(OH) D concentration below 50 nmol/L or 20 ng/mL) [23]. An adequate supplementation of calcium and vitamin D is essential, especially in patients treated with steroids [7]. Due to insufficient data, it does not recommend the routine measurement or supplementation of vitamins A, E and K in the absence of chronic liver disease. According to ESPGHAN, folic acid supplementation (1 mg daily or 5 mg weekly) is recommended in children with IBD treated with methotrexate (MTX). Vitamin B12 supplementation is recommended for children and adolescents with IBD and symptoms of vitamin B12 deficiency [23].

Our study was primarily conducted to assess the extent of dietary supplement intake among children with inflammatory bowel disease compared to a group of children without IBD. In addition, our other objectives included revealing the factors that influence the choice of individual dietary supplements, such as gender, nutritional status, diet and comorbidities, as well as understanding the reasons that determine the use of dietary supplements in children with IBD and also in children with intestinal dysfunction.

## 2. Materials and Methods

### 2.1. Study Design

The subjects were divided into two groups: an experimental group consisting of children with inflammatory bowel disease and a control group consisting of children with functional intestinal disorders.

**Figure 1:**
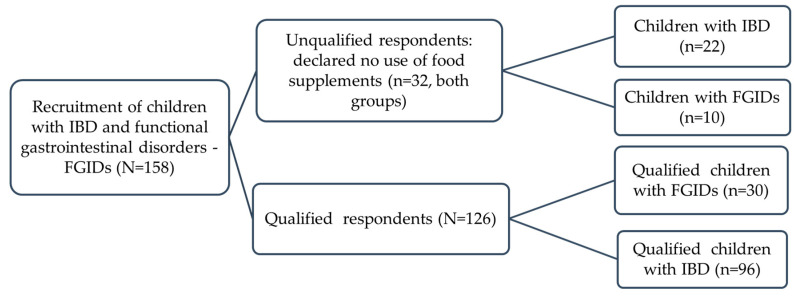
Study design. IBD—inflammatory bowel disease; FGIDs—functional gastrointestinal disorders.

The study group consisted of 126 children with IBD aged 4–18 years hospitalized consecutively in the 2nd Department of Pediatrics, Gastroenterology and Nutrition at the University Hospital in Wrocław and in the Department of Pediatrics, Gastroenterology and Nutrition at the University Children’s Hospital in Krakow. The control group consisted of children diagnosed with functional gastrointestinal disorders (16 children with functional constipation, 10 children with functional dyspepsia and 4 children with irritable bowel syndrome). Children from the control group were mainly hospitalized in Wrocław and Kraków and qualified for the study based on age and disease.The study was conducted from May 2022 to September 2023. The study was a prospective study. The participants’ parents were given a questionnaire to complete. A study design is shown in Figure 1.

Using the most recent data on the age structure of the population [24] and data on the incidence of IBD [25] and FGIDs (mainly constipation was taken into account in the calculations among the youngest part of the population [26]), and assuming a confidence level of 95% and allowing for an error of +/−10%, the minimum sample size was shown to be 97 individuals. The minimum sample size was calculated from the formula [27]:

$$n=\frac{n}{1+\frac{z^{2}\times \hat{p}\left(1-\hat{p}\right)}{\varepsilon^{2}N}}$$

*n*—sample size; *N*—population size; *z*—z score; *ε*—margin of error; $\hat{p}$—population proportion.

The respondents were then given a questionnaire to complete. Written informed consent was obtained from all participants, who were informed that the study was approved by the Bioethics Committee of the Medical University of Wrocław (KBE 777/2021). Parents of children under 16 and children over 16 agreed to participate in the study.

### 2.2. Survey Questionnaire

The study was conducted without supervision and without an interviewer. The survey was addressed to both mothers and fathers. An original, anonymous survey was used for the study. The survey questionnaire was subjected to a validation process, which resulted in a Cronbach’s coefficient of 0.7. The survey included 25 questions regarding the frequency, type and reasons for using or not using dietary supplements.

In the survey, we also asked about comorbidities and the type of diet currently followed. In the question on the type of supplemented ingredients, we included herbs and probiotics, which in some European countries, including Poland, are dietary supplements. The survey included single-choice questions, multiple-choice questions, and open questions with short answers. One question was constructed as a closed question in tabular form with the possibility of one answer on each line.

### 2.3. Anthropometric Measurements and the Assessment of Children’s Nutritional Status

Height was measured using a height meter (accurate to 0.1 cm), and body weight (accurate to 0.1 kg) was measured with a medical electronic personal scale. This was intended to obtain the highest degree of reliability for the results. When measuring both body weight and height, the standards of anthropometric tests were followed. Therefore, the measurement devices were placed on a hard, stable surface. Additionally, during the height assessment, the patient stood stably, with a straight back and head, without shoes, exactly under the height meter ruler. To assess the nutritional status of children, BMI values were used (BMI = body weight [kg]/body height [m^2^]). The aim of the nutritional assessment was to identify children with under- or overweight, which was then used in the analyses. The results were compared to the growth standards developed by the World Health Organization (WHO), depending on the child’s gender and age. We compared the BMI results with the current WHO-recommended BMI charts for age, which allowed us to establish the limits of underweight, normal weight and excess weight in children, which included overweight and obesity [28].

### 2.4. Statistical Methods

Software programs such as Microsoft Excel 2019 and Jamovi Version 4.1 were used to statistically process the results. The Chi2 test was used to analyze the responses to the survey in relation to the selected factors. A *p*-value of ≤0.05 (α = 0.05) was taken as the level of significance.

## 3. Results

### 3.1. Study Group Characteristics

The study group included 158 children attending two gastroenterology departments, of whom 118 (74.7%) were diagnosed with IBD (74 CD and 44 UC) and 40 (25.3%) with FGIDs. The questionnaire was completed by parents (89.2% were mothers and 10.8% were fathers), which was included in the group characteristics. The exact characteristics of the entire study group are presented in Table 1.

### 3.2. Assessment of Dietary Supplement Use among the Study Groups

**Figure 2:**
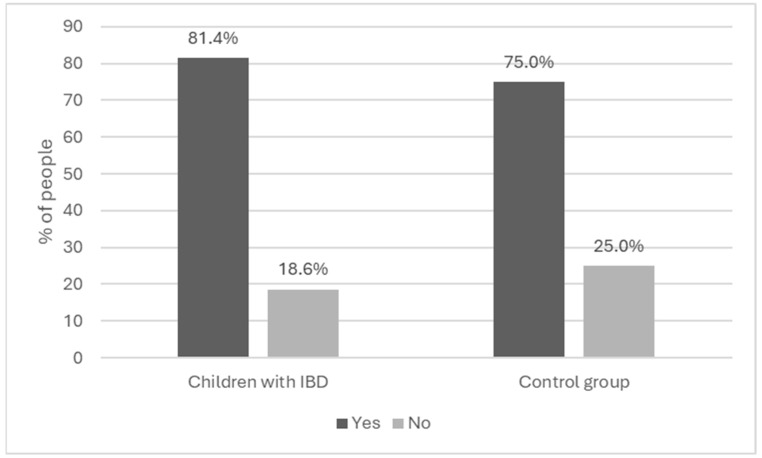
Use of dietary supplements N = 126.

Dietary supplements were given to 81.4% of the children with IBD and 75.0% of the children in the control group (Figure 2).

In the group of children with IBD, 18.6% did not use dietary supplements; 25.0% of the children from the control group did not use supplements. According to the parents, the reasons for not taking supplements were that medical staff did not recommend it (19 parents [86.4%]) and that the parents believed their child’s diet did not require it (3 parents [13.6%]). Of the 10 parents of children in the control group, 5 (50%) declared that no one had recommended supplementation for their child’s diet, 3 (30.0%) declared that their child’s diet did not require supplementation, 1 (10.0%) declared contraindications to supplementation, and 1 (10.0%) did not believe in the use of dietary supplements. In the group of children with IBD, statistical significance (*p* = 0.021) was observed in relation to the use of supplementation and disease stage. The definition of disease stage included remission (absence of symptoms in the patient) or an active disease state (occurrence of disease symptoms). This was determined using the Pediatric Crohn’s Disease Activity Index (PCDAI) [29] and the Pediatric Ulcerative Colitis Activity Index (PUCAI) [30] scores which define the period of IBD in the pediatric population. Other data are included in Table 2.

### 3.3. Use of Dietary Supplements and Type of Diet

Among the eligible children with IBD, 40.7% of their parents declared that they follow a special diet with their children. In the control group, 55% of parents declared that their children followed no diet. All results are shown in Table 3.

### 3.4. Declaration of Consuming Different Types of Dietary Supplements among the Study Groups

Among the parents of children with IBD, 84.4% declared that they supplemented with vitamins and 51% with probiotics. A similar result was obtained in the control group, in which the same types of supplements were consumed by 96.7% and 50%, respectively. A similar result was also obtained for the declaration of intake of vitamin C and D supplements. In the group of children with IBD, these vitamin supplements were taken by 30.2% and 95.8%, respectively, and in the control group by 33.3% and 96.7%, respectively. The intake of iron supplements was declared on 36.5% of the surveys from children with IBD and from 20.0% of the control group. The consumption of calcium supplements in the group of children with IBD was indicated by 26.0% of parents and by 10.0% of those in the control group. The consumption of magnesium supplements was declared by 17.7% of the group with IBD and by 20% of the group with functional gastrointenstinal disorders. Other results regarding the intake of individual dietary supplements are presented in Table 4.

### 3.5. Relationship between Supplementation Use and Gender

We did not notice any differences between boys and girls in the use of particular types of dietary supplements. The results of this analysis are presented in Table 5.

### 3.6. Relationship between Dietary Supplement Intake and Nutritional Status of Children

Vitamin B12 was most frequently supplemented among children with normal nutritional status in both groups (16.7% and 10%). Folic acid was supplemented more frequently in IBD patients with normal nutritional status (11.4%). Statistically significant differences were observed in its intake in children with different nutritional statuses (*p* = 0.023). In the group of children with IBD, 13.5% of parents whose children had a normal nutritional status declared that their children took vitamin B6 supplements, and in the group of children without IBD, it was 13.3%. Vitamins A and E were most often given to children with IBD and a normal nutritional status (8.3% each). Vitamin C supplements were consumed by 21.9% of the children in the study group and 26.7% of those in the control group. Vitamin D was supplemented in the group of children with IBD and in the control group with normal nutritional status at 75.0 and 73.3%, respectively. Calcium was most frequently supplemented by children with normal nutritional status and IBD, while 6.7% of children from the control group were underweight (*p* = 0.049). Iron supplements were most frequently taken by children with a normal nutritional status in both groups (26.0% and 16.7%, respectively). Zinc and magnesium were the most frequently supplemented in the children with IBD and a normal nutritional status. Selenium supplementation was declared by 5.2% of parents of children with IBD and a normal nutritional status and by 3.3% of parents of the control children. All the relationships are shown in Table 6.

### 3.7. Relationship between Dietary Supplement Intake and Current Diet

Vitamin B12 supplements were taken by 12.5% of children with IBD who followed a diet tailored to their individual tolerance and by 13.3% of the subgroup not on any diet and with functional bowel disorders. Vitamin B9 was supplemented by 11.5% of children with IBD and 10% of the control group not following any diet. Vitamin A was supplemented in the group of children with IBD most frequently among the subgroup following a tolerance-adapted diet (7.3%), while in the control group, vitamin A supplementation was declared by one person who did not follow any diet. Vitamins C and D were supplemented by 18.8% and 47.9%, respectively, of children with IBD following a different tolerance diet, while in the control group, these vitamins were most often supplemented in the group with no dietary restrictions (26.7 and 73.3%). In the group of children with IBD and those supplementing vitamin E, a statistical significance was demonstrated at *p* = 0.040. All minerals were most frequently supplemented in children with IBD declaring the use of another diet adapted to their tolerance; in the group of children with functional disorders, these minerals were most frequently supplemented by children not following any diet. There was statistical significance (*p* = 0.006) in the group of children with IBD regarding the response to iron supplementation relative to their current diet. Other results are presented in Table 7.

### 3.8. Dietary Supplements for Specific Diseases and the Reasons for Their Use

Among children with IBD, a significantly higher percentage of probiotic use was noted for food intolerances (18.7%) than among children without IBD (5.0%) (*p* = 0.026). Children with IBD and concomitant food allergies were more likely to take zinc supplements (*p* = 0.028). Also, children with IBD were more likely than the controls to take mineral supplements (*p* = 0.060), including iron with coexisting anemia (*p* < 0.01). With coexisting GERD, the children in the control group were more likely to take probiotics (*p* = 0.020) and magnesium (*p* = 0.033) than children with IBD. The most important relationships are shown in Table 8.

### 3.9. Reasons for Using Dietary Supplements

Among children with IBD (80.2%) and those in the control group (33.3%), the reason for dietary supplementation was a doctor’s recommendation. In the IBD group, 42.7% of children took supplements due to nutrient deficiencies and to replenish deficiencies. In the control group, 43.3% took supplements for the same reason. All results of the statistical analysis can be found in Table 9.

## 4. Discussion

There is a growing trend worldwide among different populations to consume dietary supplements [31,32,33,34,35,36,37,38]. This is mainly due to the belief that they can replace a balanced diet, but also to the unregulated status of these products in some countries, which means that some populations have easy access to dietary supplements. This trend also applies to healthy children [39,40,41,42,43] and those with various diseases, such as inflammatory gastrointestinal disease [44]. Our study showed that the use of dietary supplements was more frequently declared by parents of children with IBD, suggesting that the disease is one of the determinants of supplement use. This suggests monitoring the intake of dietary supplements among children with chronic diseases, because the ingredients of dietary supplements may interact with medications and reduce the effectiveness of therapy. However, as our study showed, the disease status (remission or active form) may also determine the use of dietary supplements among children with CD or UC, as it is associated with concerns that in the active form of the disease these children may develop nutritional deficiencies. In the work of other authors [45], it was found that among adolescents with IBD, complementary alternative medicine (CAM), which includes dietary supplements, was more frequently used in the inactive and mild disease state, without any division into CD and UC. The results of both studies are similar, which also highlights the fact that people with IBD should follow the recommendations for supplementation not only during the period of disease remission but also during periods of exacerbation.

In several studies [46,47] showing what type of diet is used in the pediatric population with IBD, the most common diet used among adolescents was a lactose-free, gluten-free diet, without dietary restrictions or excluding other foods. The use of lactose-free diets, or other diets excluding a given ingredient or product, is mainly due to the fact that these children may have a non-immunological reaction of the body to the consumed food, such as lactose intolerance or other intolerances, e.g., non-celiac intolerance to gluten/wheat or other nutrients. The use of such diets may also result from the beliefs of parents who, having access to various information in books or on the Internet, start using such diets without consulting a doctor or dietician. It should be emphasized here that such behavior may be the cause of nutritional deficiencies in children and adolescents with IBD. The results of our study confirmed our hypothesis that most children in the control group will not follow any dietary restrictions, and children with IBD will follow a diet adjusted to tolerance. This is mainly due to the severity of the disease and individual tolerance of products, foods or their ingredients, especially among children and adolescents with IBD.

Vitamins, minerals and probiotics are commonly added to various types of dietary supplements. This is mainly due to the easy access to them and the belief that one’s diet does not provide the appropriate amount of vitamins and minerals because access to fresh vegetables and fruits is limited, especially in the winter. There is also a belief that excess vitamins have a health-promoting effect on the body. Similar results regarding the use of multivitamins as dietary supplements have been obtained by other authors [45,46], which confirms that this is the most commonly used group of supplements. Among the supplemented minerals in our study, iron was more often taken by children with IBD, especially in the case of the co-occurrence of anemia, which is a consequence of the disease. Our results confirmed iron supplementation in children with IBD and concomitant anemia, which is in line with the recommendations of ESPEN [7], ECCO [22] and NASPGHAN [48], which all recommend iron supplementation when anemia is diagnosed in children and adults with IBD. Also, recommendations for oral iron supplementation in the pediatric population in the case of anemia in IBD were developed by the SIGENP IBD Working Group [49] and Portuguese Society of Pediatric Gastroenterology, Hepatology and Nutrition (SPGP)—Anemia–IBD Working Group [50].

In our study, probiotics were also frequently consumed and used in both groups of children. Probiotics can also be one of the elements of CAM, being in the form of dietary supplements and therefore easily accessible to consumers. At this point, it should be emphasized that not all probiotics have the same effect and also not for all diseases. Therefore, the education of parents by a doctor or dietician on the use of probiotics is extremely important.Another result of our study is the common use of dietary supplements containing herbs, which is one of the complementary and alternative medicine (CAM) approaches.

This may be due to the wide availability of such preparations in pharmacies and stores. Another problem with CAM is the fact that many patients do not disclose the use of herbal medicines. Therefore, it is necessary to strive to obtain certainty about the efficacy and safety of dietary supplements with herbs and to develop recommendations based on reliable scientific evidence regarding their use in the treatment of IBD.

In a group of Polish children with inflammatory bowel disease, we observed a difference between folic acid supplementation and the children’s nutritional status. This is an important result because it draws attention to the need for supplementation in children with a different nutritional status, IBD—especially Crohn’s disease [51]—and those treated with methrotrexate, which can lead to folic acid deficiency [52]. Monitoring folic acid levels in children with IBD and increased body weight is recommended because it can lead to anemia and can impact homocysteine levels [53]. According to ESPGHAN [23] recommendations, a significant folic acid deficiency is rare in children with IBD and the need for folic acid in children with CD has not been determined. We agree that supplementation is important in the case of deficiency.

In the control group, body mass index conditioned calcium and zinc supplementation. It seems important to consider calcium supplementation in children with obesity, as the current evidence suggests an effect of calcium in preventing obesity in this population [54,55]. According to ESPGHAN [23], calcium should not be supplemented in children with IBD without any medical indication, but only at times of increased low calcium intake and a risk of osteoporosis. We also recommend that attention be paid to zinc supplementation, as children with excess body weight may have abnormal blood zinc concentrations [56]. This is all the more important because zinc supplementation has been shown to have a beneficial effect on reducing anthropometric measurements, inflammatory markers, insulin resistance and appetite in obese individuals [57]. According to ESPGHAN recommendations, zinc supplementation is only recommended in cases of proven zinc deficiency [23]. Statistically significant in our study in the case of coexisting intolerances was the consumption of probiotics, which in Poland are also available in the form of dietary supplements. As with vitamins and minerals, there is a belief that supplementation with any probiotic is necessary for the body. However, there are no clear recommendations regarding supplementation in the case of CD, while more convincing scientific evidence exists for probiotic supplementation in UC [58,59]. Probiotics have been shown to improve lactose metabolism in cases of intolerance [60,61] and to reduce symptoms [62] and the development of food intolerance itself [63]; therefore, if this condition is diagnosed, supplementation may be considered. The ESPGHAN scientific position [64] clearly defines that there is no adequate evidence for the use of probiotics in IBD. However, they are recommended for the treatment or prevention of other gastrointestinal problems, such as functional constipation or diarrhea.

In children with concomitant allergies, an adequate supply of zinc should also be considered, as increased total and allergen-specific IgE levels have been shown to be significantly associated with reduced zinc levels [65], and zinc may combat the atopic march through appropriate food fortification or supplementation [66].

In the present study, supplementation with probiotics was significant in children with IBD and co-occurring GERD. It may seem reasonable to use probiotic therapy in reflux esophagitis, as esomeprazole in combination with probiotics (*B. subtilis* and *E. faecium*) has a beneficial effect on RE treatment and patient management [67] and can reduce the rate of intestinal dysbiosis among children treated with PPIs [68]. This implies that supplementation with appropriate probiotic strains may be beneficial for both alleviating GERD symptoms and improving the microbiota altered by IBD.

The results of our study show differences between the motives of parents of healthy children and children with IBD. The reason for using dietary supplements among the control group coincides with other studies’ findings [69,70,71,72], which may indicate parental concern that the child’s diet is not properly balanced. Among children with IBD, this result may indicate adherence to medical advice, as it is mainly doctors who decide on the supplements that are used by this group of patients. Other reasons for supplementation were to supplement deficiencies, prevent diarrhea and improve immunity. These reasons are often the main reason for giving dietary supplements and often without consulting a doctor.

The study was limited by the number of participants, because inflammatory bowel disease is a rare disease and its prevalence varies across countries. The number of children with FGIDs may also be limited, and parents of such children may not always consent to participate in the study. Therefore, another limitation of our study was the consent of the patients’ parents to participate in the study. Such factors should be taken into account when planning similar studies in the future.

## 5. Conclusions

Dietary supplements are used in children with functional bowel disease and inflammatory bowel disease. However, there are differences in the use of dietary supplements between these groups, which may be due to several factors, such as disease activity, nutritional status, current diet and comorbidities. Particular attention should be paid to children with inflammatory bowel disease and other comorbidities, as this group is often at risk of nutritional deficiencies related to dietary restrictions. In such a situation, an appropriately tailored diet and supplementation support may have a beneficial therapeutic effect. When diet does not cover nutrient requirements, supplementation is essential and has beneficial effects for improving overall health. Also, the provision of probiotics, either in the form of dietary supplements or with natural foods, can improve the function of the whole organism, including the immune system, whose work may be disrupted in the case of malnutrition. Supplementation with a suitable probiotic preparation will be important in situations of diarrhea, the consequence of which may be nutrient and mineral deficiencies.

It is important to ensure that IBD patients are properly educated about dietary supplements, as it is possible that they may be taken in excess and cause adverse side effects. In this situation, we recommend special caution in the use of dietary supplements by patients, which should be monitored by a doctor or dietician once an individual supplementation and nutrition plan has been established. Another conclusion of the study is that further research is needed to demonstrate the frequency of intake, reasons for intake and type of dietary supplements consumed by paediatric patients with inflammatory bowel disease. This is important in the context of future recommendations for inflammatory bowel disease, and the literature review shows little information on supplement intake among children with inflammatory bowel disease. This would provide new scientific evidence and specific recommendations for supplementation in inflammatory bowel disease.

## Figures and Tables

**Table 1:** Characteristics of the group N = 158.

Characteristics	Total N = 158 N (%)	Children with IBD N = 118		Control Group N = 40 N (%)	*p*-Value
		Crohn Disease N (%)	Ulcerative Colitis N (%)		
Sex of children					<0.01
Girls	76 (48.1)	33 (27.9)	30 (25.4)	13 (32.5)	
Boys	82 (51.9)	41 (72.1)	14 (74.6)	27 (67.5)	
Age [years]	13.0 ± 3.7	13.8 ±3.3		10.7 ± 4.0	<0.01
BMI [kg/m^2^]					<0.01
Girls	18.42 ± 3.55	18.74 ± 3.26	19.18 ± 3.72	17.34 ± 3.66	
Boys	18.76 ± 3.68	19.19 ± 3.05	19.10 ± 3.90	17.98 ± 4.09	
Nutritional status					0.875
Low weight	20 (12.6)	14 (11.9)		6 (15.0)	
Normal weight	118 (74.8)	89 (75.4)		29 (72.5)	
Overweight/obese	20 (12.6)	15 (12.7)		5 (12.5)	
Residence					<0.01
Village	57 (36.1)	40 (33.9)		17 (42.5)	
City	91 (63.9)	78 (66.1)		23 (57.5)	
Sex of the parent completing the questionnaire					0.858
Female	141 (89.2)	105 (88.9)		36 (90.0)	
Male	17 (10.8)	13 (11.1)		4 (10.0)	
Coexisting diseases *					0.171
Intolerance (gluten, lactose)	38 (22.8)	32 (27.1)		6 (15.0)	
Food allergy	26 (16.4)	20 (16.9)		6 (15.0)	
Anemia	31 (19.6)	30 (25.4)		1 (2.5)	
Thyroid diseases	4 (2.5)	4 (3.4)		-	
Low stature	10 (6.3)	10 (8.5)		-	
GERD	17 (10.7)	9 (7.6)		8 (20.0)	
Type of diet					<0.01
CDED	20 (12.7)	20 (16.9)		-	
Other special diets	71 (44.9)	60 (50.8)		11 (27.5)	
Lactose-free	18 (11.4)	14(11.9)		4 (10.0)	
Gluten/wheat-free	3 (1.9)	2 (1.7)		1 (2.5)	
Specific carbohydrate	1 (0.6)	1 (0.8)		-	
Another individual diet	46 (29.1)	41 (34.7)		5 (12.5)	
Without dietary restrictions	67 (42.4)	38 (32.2)		29 (72.5)	
Diseases phass					<0.01
Remmission	-	58 (49.1)	25 (21.2)		
Activ	-	16(13.5)	19 (16.2)	-	

* Multiple-choice question; CDED, Crohn’s Disease Exclusion Diet; GERD, Gastroesophageal Reflux Disease.

**Table 2:** Disease stage and use of dietary supplements in group of children with IBD N (%).

	Remission		Activ		All		*p*-Value
	N	%	N	%	N	%	
Crohn disease	58	69.9	16	45.7	74	62.7	0.021
Ulcerative collitis	25	30.1	19	54.3	44	37.3	

**Table 3:** Relationship between type of diet and intake of dietary supplements.

Use of Supplements among Whole Group	Children with IBD N = 96			*p*-Value	Control Group N = 30		*p*-Value
	CDED Diet N (%)	Special Diet N (%)	Without Dietary Restrictions N (%)		Special Diet N (%)	Without Dietary Restrictions N (%)	
Yes	15 (12.7 )	48 (40.7)	33 (28.0 )	0.483	8 (20.0 )	22 (55.0 )	0.838
No	5 (4.2 )	12 (10.2)	5 (4.2 )		3 (7.5 )	7 (17.5 )	

CDED—Crohn’s Disease Exclusion Diet.

**Table 4:** Use of dietary supplements among participations in the study group.

Characterictics of Supplement	Children with IBD N (%)	Control Group N (%)	*p*-Value
Kind of dietary supplements used			
Vitamins	81(84.4)	29 (96.7)	0.018
Minerals	47 (48.9)	10 (33.3)	0.005
Multicomplex	18 (18.7)	2 (6.7)	0.278
Herbs	5 (5.2)	2 (6.7)	0.013
Proteins	3 (3.1)	1 (3.3)	0.070
Omega-3	21 (21.8)	6 (20.0)	0.469
Probiotics	49 (51.0)	15 (50.0)	0.214
Vitamins *			
Vitamin B6	16 (16.7)	6 (20.0)	0.192
Vitamin B9	16 (16.7)	4 (13.3)	0.516
Vitamin B12	19 (19.8)	5 (16.7)	0.786
Vitamin A	11 (11.4)	1 (3,3)	0.161
Vitamin C	29 (30.2)	10 (33.3)	0.473
Vitamin D	92 (95.8)	29 (96.7)	0.824
Vitamin E	9 (9.4)	1 (3.3)	0.228
Minerals *			
Ca	25 (26.0)	3 (10.0)	0.051
Fe	35 (36.5)	6 (20.0)	0.042
Mg	17 (17.7)	6 (20.0)	0.872
Zn	11 (11.4)	4 (13.3)	0.773
Se	5 (5.2)	1 (3.3)	0.654

* Multiple-choice question.

**Table 5:** Declaration of use of dietary supplements according to gender N (%).

Characterictics of Suplement	Children with IBD N (%)		Control Group N (%)		*p*-Value
	Girls	Boys	Girls	Boys	
Kind of dietary supplements used					
Vitamins	40 (41.7)	41 (42.7)	16 (53.3)	13 (43.3)	0.386
Minerals	25 (26.0)	22 (22.9)	6 (20.0)	4 (13.3)	0.682
Multicomplex	8 (8.3)	10 (10.4)	2 (6.7)	0.0	0.816
Herbs	2 (2.1)	3 (3.1)	1 (3.3)	1 (3.3)	0.604
Proteins	2 (2.1)	1 (1.0)	1 (3.3)	0.0	0.357
Omega-3	9 (9.4)	12 (12.5)	4 (13.3)	2 (6.7)	0.619
Probiotics	26 (27.1)	23 (23.9)	9 (30.0)	6 (20.0)	0.598
Vitamins *					
Vitamin B6	7 (23.3)	9 (30.0)	3 (10.0)	3 (10.0)	0.474
Vitamin B9	9 (30.0)	7 (7.3)	3 (10.0)	1 (3.3)	0.457
Vitamin B12	7 (7.3)	12 (12.5)	3 (10.0)	2 (6.7)	0.243
Vitamin A	4 (4.2)	7 (7.3)	1 (3.3)	0.0	0.435
Vitamin C	14 (14.6)	15 (15.6)	3 (10.0)	7 (23.3)	0.186
Vitamin D	46 (47.9)	46 (47.9)	16 (53.3)	13 (43.3)	0.207
Vitamin E	4 (4.2)	5 (5.2)	1 (3.3)	0.0	0.337
Minerals *					
Ca	12 (12.5)	13 (13.5)	3 (10.0)	0.0	0.886
Fe	21 (21.9)	14 (14.6)	3 (10.0)	3	0.337
Mg	7 (23.3)	10 (10.4)	4 (13.3)	3 (10.0)	0.629
Zn	3 (3.1)	8 (8.3)	3 (10.0)	2 (6.7)	0.306
Se	2 (2.1)	3 (3.1)	1 (3.3)	1 (3.3)	0.905

* Multiple-choice question.

**Table 6:** Relationship between children’s BMI and the supplementation of individual minerals and vitamins N (%).

	Children with IBD N (%)				Control Group N (%)			
	Underweight	Normal	Obesity	*p*-Value	Underweight	Normal	Obesity	*p*-Value
Vitamin B12	2 (2.1)	16 (16.7)	1 (1.0)	0.563	2 (6.7)	3 (10.0)	0.0	0.276
Vitamin B9	0.0	11 (11.4)	5 (5.2)	0.023	2 (6.7)	2 (6.7)	0.0	0.149
Vitamin B6	2 (2.1)	13 (13.5)	1 (1.0)	0.697	2 (6.7)	4 (13.3)	0.0	0.397
Vitamin A	2 (2.1)	8 (8.3)	1 (1.0)	0.639	1 (3.3)	0.0	0.0	0.075
Vitamin C	2 (1.6)	21 (21.9)	6 (6.25)	0.242	1 (3.3)	8 (26.7)	1 (3.3)	0.715
Vitamin D	9 (9.3)	72 (75.0)	11 (11.4)	0.224	5 (16.7)	22 (73.3)	2 (6.7)	0.854
Vitamin E	1 (1.0)	8 (8.3)	0.0	0.490	1 (3.3)	0.0	0.0	0.075
Ca	1 (1.0)	21 (21.9)	3 (3.1)	0.460	2 (6.7)	1 (3.3)	0.0	0.049
Fe	5 (5.2)	25 (26.0)	5 (5.2)	0.560	1 (6.7)	5 (16.7)	0.0	0.762
Zn	1 (1.0)	8 (8.3)	2 (2.1)	0.830	2 (6.7)	1 (3.3)	1 (3.3)	0.030
Mg	1 (1.0)	15 (15.6)	1 (0.8)	0.481	2 (6.7)	3 (10.0)	1 (3.3)	0.215
Se	0.0	5 (5.2)	0.0	0.457	1 (3.3)	0.0	0.0	0.075

**Table 7:** Type of diet and use of mineral and vitamin supplementation.

Use of Supplement Vitamins/Minerals	Children with IBD N (%)			*p*-Value	Control Group N (%)		*p*-Value
	CDED Diet	Other Diet	Without Dietary Restrictions		Special Diet	Without Dietary Restrictions	
Vitamin B12	4 (4.2)	12 (12.5)	3 (3.1)	0.161	1 (3.3)	4 (13.3)	0.712
Vitamin B9	3 (3.1)	11 (11.5)	2 (2.1)	0.126	1 (3.3)	3 (10.0)	0.935
Vitamin B6	3 (3.1)	12 (12.5)	1 (1.0)	0.018	1 (3.3)	5 (16.7)	0.536
Vitamin A	3 (3.1)	7 (7.3)	1 (1.0)	0.106	0.0	1 (3.3)	0.540
Vitamin C	5 (5.2)	18 (18.8)	6 (6.3)	0.170	2 (6.7)	8 (26.7)	0.559
Vitamin D	15 (15.6)	46 (47.9)	31(32.3)	0.622	7 (23.3)	22 (73.3)	0.092
Vitamin E	2 (2.1)	7 (7.3)	0.0	0.040	0.0	1 (3.3)	0.540
Fe	7 (7.3)	23 (24.0)	5 (5.2)	0.006	2 (6.7)	4 (3.3)	0.645
Ca	4 (4.2)	16 (16.7)	5 (5.2)	0.186	1 (3.3)	2 (6.7)	0.783
Mg	5 (5.2)	9 (9.4)	3 (3.1)	0.121	2 (6.7)	4 (13.3)	0.645
Zn	2 (2.1)	6 (6.3)	3 (3.1)	0.831	1 (3.3)	3 (10.0)	0.935
Se	2 (2.1)	3 (3.1)	0.0	0.107	0.0	1 (3.3)	0.540

**Table 8:** Frequency of intake of individual components according to the presence of comorbidities.

Diseases	Children with IBD N (%)	Control Group N (%)	*p*-Value
Intolerance (gluten, lactose) probiotics	18 (18.7)	2 (5.0)	0.026
Food allergy Zn	5 (5.2)	1 (2.5)	0.028
Anemia Minerals Fe	17 (17.5) 16 (16.6)	1 (2.5) 1 (2.5)	0.060 <0.01
GERD Probiotics Mg	7 (7.3) 2 (2.1)	7 (17.5) 4 (10.0)	0.020 0.033

**Table 9:** Reasons to use dietary supplements in both groups.

Reason	Children with IBD N = 96 N (%)		Control Group N = 30 N (%)		*p*-Value
	Yes	No	Yes	No	
Weight loss	1 (1.0)	95 (98.9)	0	30 (100.0)	0.575
Improvement of skin condition	6 (6.25)	90 (93.7)	0	30 (100.0)	0.161
Improvement of physical and mental condition	3 (3.1)	93 (96.9)	2 (6.7)	28 (93.3)	0.386
Compensation for nutrient deficiencies	41 (42.7)	55 (57.3)	13 (43.3)	17 (56.7)	0.952
Physician’s recommendation	77 (80.2)	19 (19.8)	10 (33.3)	20 (66.7)	<0.001
Recommendation by a dietician	5 (5.2)	91 (97.8)	0	30 (100.0)	0.202
Improving immunity	16 (16.7)	80 (83.3)	10 (33.3)	20 (66.7)	0.049
Prevention of diarrhea	10 (10.4)	86 (89.6)	2 (6.7)	28 (93.3)	0.011
Improvement of intestinal microbiota	19 (19.8)	77 (80.2)	8 (26.7)	22 (73.3)	0.423

## Data Availability

Data are contained within the article.
